# Immune Cell Interactions and Immune Checkpoints in the Tumor Microenvironment of Gastric Cancer

**DOI:** 10.3390/ijms26031156

**Published:** 2025-01-29

**Authors:** Andreea-Raluca Cozac-Szőke, Dan Alexandru Cozac, Anca Negovan, Andreea Cătălina Tinca, Alexandra Vilaia, Iuliu-Gabriel Cocuz, Adrian Horațiu Sabău, Raluca Niculescu, Diana Maria Chiorean, Alexandru Nicușor Tomuț, Ovidiu Simion Cotoi

**Affiliations:** 1 Doctoral School of Medicine and Pharmacy, George Emil Palade University of Medicine, Pharmacy, Science and Technology of Targu Mures, 540142 Targu Mures, Romania; andreea-raluca.szoke@umfst.ro (A.-R.C.-S.); adrian-horatiu.sabau@umfst.ro (A.H.S.); raluca.niculescu@umfst.ro (R.N.); diana.chiorean@umfst.ro (D.M.C.); 2 Pathophysiology Department, George Emil Palade University of Medicine, Pharmacy, Science, and Technology of Targu Mures, 540142 Targu Mures, Romania; andreea-catalina.tinca@umfst.ro (A.C.T.); iuliu.cocuz@umfst.ro (I.-G.C.); ovidiu.cotoi@umfst.ro (O.S.C.); 3 Pathology Department, Mures Clinical County Hospital, 540011 Targu Mures, Romania; 4 Emergency Institute for Cardiovascular Diseases and Transplantation Targu Mures, 540142 Targu Mures, Romania; 5 Department of Clinical Science-Internal Medicine, George Emil Palade University of Medicine, Pharmacy, Science, and Technology of Targu Mures, 540142 Targu Mures, Romania; ancanegovan@yahoo.com; 6 Department of Infectious Diseases I, Doctoral School of Carol Davila University of Medicine and Pharmacy, 050474 Bucharest, Romania; alexandra.vilaia@gmail.com; 7 Faculty of Medicine, George Emil Palade University of Medicine, Pharmacy, Science, and Technology of Targu Mures, 540142 Targu Mures, Romania; nicusortomut19@gmail.com

**Keywords:** TILs, TAMs, PD-L1, Siglec-15, tumor microenvironment, immunotherapy

## Abstract

Gastric cancer (GC) ranks as the fifth most prevalent malignant neoplasm globally, with an increased death rate despite recent advancements in research and therapeutic options. Different molecular subtypes of GC have distinct interactions with the immune system, impacting the tumor microenvironment (TME), prognosis, and reaction to immunotherapy. Tumor-infiltrating lymphocytes (TILs) in the TME are crucial for preventing tumor growth and metastasis, as evidenced by research showing that patients with GC who have a significant density of TILs have better survival rates. But cancer cells have evolved a variety of mechanisms to evade immune surveillance, both sialic acid-binding immunoglobulin-like lectin 15 (Siglec-15) and Programmed Death-Ligand 1 (PD-L1) playing a pivotal role in the development of an immunosuppressive TME. They prevent T cell activation and proliferation resulting in a decrease in the immune system’s capacity to recognize and eliminate malignant cells. These immune checkpoint molecules function via different but complementary mechanisms, the expression of Siglec-15 being mutually exclusive with PD-L1 and, therefore, providing a different therapeutic approach. The review explores how TILs affect tumor growth and patient outcomes in GC, with particular emphasis on their interactions within the TME and potential targeting of the PD-L1 and Siglec-15 pathways for immunotherapy.

## 1. Introduction

Gastric cancer (GC) is the fifth most common malignant tumor diagnosed worldwide, characterized by a high mortality rate despite the recent research and strategies developed for cancer treatment [1]. According to the Lauren classification, GC has four subtypes: intestinal, diffuse, mixed, and unclassified [2]. The Cancer Genome Atlas Research Network proposed a novel molecular classification of GC: Epstein–Barr virus-positive (EBV), microsatellite instability (MSI), chromosomal instability (CIN), and genomically stable (GS) [3].

The molecular classification of GC has provided a better understanding of the disease’s heterogeneity and its relationship with the immune system. The immune system interacts differently with different molecular subtypes of GC, affecting the tumor microenvironment (TME), prognosis, and response to treatments, including immunotherapy [4,5].

Since immune cells of the innate and adaptive immune systems are part of the TME, immune system reactions can potentially eliminate cancer cells or modify their characteristics and functions [6]. But to evade immune surveillance, cancer cells have developed various strategies, including weak points in the antigen-presenting cells, the overexpression of negative regulatory pathways, and the chemoattraction of immunosuppressive cells in the TME. This has limited the ability of immune cells to perform their functions and inhibited the immune system’s capacity to fight cancer [7].

Tumor-infiltrating lymphocytes (TILs) form a major component of the immune response and significantly influence tumor progression and patient prognosis. TILs in the TME give evidence of an active immune response, which might be necessary to restrict tumor growth and metastasis. In patients with GC, it has been demonstrated that higher densities of TILs are associated with higher survival rates, indicating a protective role of TILs against tumor progression [8,9].

Programmed Death-Ligand 1 (PD-L1) interacts with the Programmed Cell Death-Protein-1 (PD-1) receptor on the surface of T cells and reduces the activity of T cells, decreasing their ability to mount an effective attack against the tumor [10].

Sialic acid-binding immunoglobulin-like lectin 15 (Siglec-15), like PD-L1, can contribute to creating an immunosuppressive TME. By interacting with its receptors on T cells, Siglec-15 can inhibit T cell activation and proliferation, reducing the immune system’s ability to target and destroy tumor cells [11].

Siglec-15 and PD-L1 function through complementary but distinct pathways [12]. PD-L1 inhibits T cell function by binding to PD-1; in contrast, Siglec-15 interacts with different receptors, such as sialic acid-binding receptors expressed on T cells to exert its immunosuppressive functions. Interestingly, Siglec-15 expression is often mutually exclusive with PD-L1 expression, suggesting that tumors that do not express PD-L1 may express Siglec-15 instead, thus serving as a potential alternative target for immunotherapy in PD-L1 negative patients [13,14].

The present review article aims to provide a better understanding of the relationship between GC, TILs, and immune checkpoint molecules PD-L1 and Siglec-15. All the discussion is integrated into the GC TME, exploring how TILs influence tumor progression and patient outcomes. A particular focus was directed to Siglec-15 and PD-L1 involvement in immune evasion and their potential targeting in immunotherapy.

## 2. TILs

Traditionally, the population of lymphocytes that infiltrate the tumor microenvironment—especially T cells and B cells—is referred to as “tumor-infiltrating lymphocytes (TILs)”. But the term “tumor-infiltrating leukocytes” or “tumor-infiltrating immune cells” refers to a wider range of immune cells than merely lymphocytes. This larger category includes various cell types involved in the tumor’s immune response, including macrophages, dendritic cells, mast cells, neutrophils, and myeloid-derived suppressor cells (MDSCs) [15,16].

In this review, the focus is primarily on T lymphocytes, B lymphocytes, natural killer (NK) cells, and tumor-associated macrophages (TAMs) and their important roles in modifying the TME, impacting tumor growth, immunological responses, and the efficacy of immunotherapy in GC.

### 2.1. T Lymphocytes

The immunological landscape of GC is influenced by distinct subsets of lymphocytes, which can lead to significant differences in TILs’ composition. In the setting of EBV-associated GC or MSI, CD8^+^ cytotoxic T lymphocytes (CTLs) are particularly important because they can directly induce apoptosis in cancer cells, and their presence is associated with better clinical outcomes [17,18]. TILs additionally comprise T-helper 17 (Th17) and regulatory T cells (Tregs), the balance between which affects the TME and the immune system as a whole [19].

T cell receptor (TCR) subunits and the key lineage markers CD8 and CD4 are used to classify T lymphocytes. The αβTCR complex gives T cells the ability to recognize peptides on their cell surface according to the major histocompatibility complex (MHC) class I (for CD8 T cells) or class II (for CD4 T cells). The γδ TCR component, on the other hand, is believed to function mainly independently of MHC classes I and II [20,21].

#### 2.1.1. CD8^+^ T Cells

CD8^+^ T or CTLs are known for their exceptional antiviral and anticancer properties. The capacity of tumor-specific CD8^+^ T cells to destroy tumor cells makes them a very important component of TILs. Evidence has demonstrated that an increased density of tumor-specific CD8^+^ is associated with a better prognosis in several types of malignancies [22].

High concentrations of cytotoxic molecules and antitumor cytokines, including interferon-γ (IFN-γ), tumor necrosis factor-α (TNF-α), perforin, and granzymes, can be produced by CTLs [23].

After their primary targets are eliminated, CTLs generate a variety of memory subsets that offer long-term protection against reinfection: stem cell-like memory T cells, central memory T cells, effector memory T cells, effector memory Rheumatoid Arthritis (RA)+ T cells, and peripheral tissue-resident memory cells (TRM) [24]. The most notable naive cells are stem cell-like memory T. They are primarily in lymph nodes, followed by spleen and bone marrow. Stem cell-like memory T cells assure their self-renewal and can divide quickly and generate inflammatory cytokines. Although central memory T cells have naive-like characteristics, they show a reduced ability to self-renew. Effector memory RA+ T cells preferentially travel to peripheral tissues and exhibit pro-inflammatory effector functions following secondary antigen contact with a cognate antigen [25,26].

TRM function in tumor immunity has gained more attention lately. These cells express CD39 and CD103, the latter forming αEβ7 complex with integrin β7, which interacts with E-cadherin. Tumor control was found to be affected by the absence of CD103^+^ CTLs or E-cadherin in cancer animal models [27,28].

In numerous solid tumors, bystander CD8^+^ T cells have also been found. These cells do not present selectivity for tumor antigens, and they are different from the group of tumor-specific CD8^+^ T cells because they exhibit a variety of phenotypes but do not express CD39. These cells are either predominantly reactive to common human pathogens such as EBV, cytomegalovirus, and influenza or demonstrate ambiguous antigen specificity [29]. While tumor-specific CD8^+^ T cells recognize and react to tumor antigens presented on MHC molecules, the bystander CD8^+^ T cells present in the TME remain in a non-functional state or significantly contribute in the local immunological microenvironment by secreting several cytokines and chemokines in a way that could be beneficial or detrimental to tumor control [30].

During research on tumor-specific CD8^+^ T cells in malignancies, there are two main consequences of tumor infiltration by the bystander CD8^+^ T cells. Firstly, the co-presence of bystanders may create confusion regarding the link between tumor-specific CD8^+^ T cells infiltration and response to immune checkpoint inhibitors since tumor-specific CD8^+^ T cells are mandatory for effective tumor destruction, and their presence is associated with a better response to immunotherapy. If non-functional bystanders outnumber tumor-specific T cells, they can alter the immune responses, and the impact of immunotherapy will be more difficult to predict. The ranging percentage of bystanders compared to tumor-specific cells CD8^+^ T cells across different tumors and individuals may explain the heterogeneity observed in response to immune checkpoint inhibitors [31,32,33,34,35].

Another aspect to be considered is the likelihood of bystander T cells to differentiate into tumor-specific CD8^+^ T cells depending on their exposure to tumor antigens. If bystander CD8^+^ T cells interact with tumor antigens in the presence of appropriate cytokines and co-stimulatory signals, they can become tumor-specific CD8^+^ T cells. This event is facilitated by the antigen cross-presentation assured by DCs or other APCS [36,37].

For this reason, such infiltration can greatly complicate investigations examining the functions of T lymphocytes in malignancies, unless their antigen specificity is determined. The tumor specificity of T cells cannot be determined only by observing the T cell’s presence in the TME using general immunohistochemical markers for T lymphocytes. It is, therefore, important to distinguish between the phenotype and quantity of tumor-specific T cells and the quantity and phenotype of the other tumor-infiltrating T cells, such as bystanders [30,31,32,33].

On the other hand, tumor-specific CD8^+^ T cells can lose their functions and adopt features of a bystander phenotype due to their exhaustion. The exhaustion is caused by chronic antigen stimulation, which results in impaired cytotoxicity, with decreased anti-tumor cytokine production. This event may elucidate why tumor-specific CD8^+^ T cells can appear in an inactive state and resemble bystander CD8^+^ T cells, although they maintain their antigen specificity at the molecular level [38,39,40].

Summarizing the reference literature, the phenotypic variability of CD8^+^ T cells results from two factors: (1) the nature of antigen stimulation of bystanders and (2) differentiation of tumor-specific CD8^+^ T cells (activation or exhaustion). These aspects have to be carefully considered when examining the immune landscape in malignancies, and further studies would be helpful to a better understanding of the pathophysiological mechanism of these cells in GC [38,39,40].

##### The Link Between Tumor-Elicited Immunosuppression and CTLs

The downregulation of human leukocyte antigen (HLA) expression on tumor cells is an essential way that malignancies use to avoid adaptive immunity seen in about 50% of solid tumors [41].

Tumor-associated antigens must be presented to CTLs via HLA class I molecules, this event being blocked when HLA is lost, enabling malignancies to evade immune detection in two ways: (1) preventing CTLs from identifying and eliminating tumor cells, and (2) decreasing tumor-associated antigens presentation, thereby inhibiting the activation of adaptive immune responses [41].

Mutations in antigen-processing components (TAP1/2, β2-microglobulin) or tumor-secreted cytokines (TGF-β) that inhibit HLA expression are common causes of this immunological escape mechanism [42].

Along with HLA downregulation, solid tumors take advantage of evolutionary defenses from immune-privileged areas, including the brain, placenta, testes, and anterior chamber of the eye. Tumors imitate these sites’ tactics by using Fas signaling to cause activated T cells to go through apoptosis and secretion of immunosuppressive molecules such as TGF-β, IL-10, and adenosine to suppress T cell and NK cell activity [43].

The placenta, one of the body’s most immunosuppressive environments, provides immunological mechanisms that tumors also use HLA-G expression and activation of the indoleamine 2,3-dioxygenase (IDO) pathway to evade the immune system. HLA-G, which is a non-classical HLA protein that suppresses T and NK cell activities and imitates placental defense mechanisms for the fetus, is upregulated in tumors. Tryptophan is removed by IDO, inhibiting T cell proliferation, resulting in an immunosuppressive TME similar to that found at the maternal–fetal interface [44,45].

In these ways, the borrowed strategies improve the immunosuppressive milieu by preventing immune cell infiltration, activation, and function, in addition to HLA loss which directly reduces antigen-specific immune cell responses [41,42,43,44,45].

#### 2.1.2. CD4^+^ T Cells

Based on their patterns of cytokine production and functions, Th17 and forkhead box protein P3 (FOXP3^+^) Tregs cells have been recently identified as two different CD4^+^ T subsets from Th1 and Th2 cells, with significant roles in GC [46].

FOXP3^+^ Treg cells are functionally immunosuppressive and are identified by the expression of FOXP3^+^ in the nucleus [46].

One of the important mechanisms through which FOXP3^+^ Tregs modulates systemic immunosuppression is the release of adenosine. Adenosine interacts with the adenosine A2A receptor (A2AR), decreasing the expression of adhesion molecules on endothelial cells and impairing the ability of CTLs to bind and migrate through the tumor vasculature. This event will prevent CTls from infiltrating the tumor and exerting their functions, leading to systemic immunosuppressive effects [47].

In local immunosuppression, they have important roles in maintaining tolerance to self-components by releasing anti-inflammatory cytokines like transforming Growth Factor-Beta (TGF-β) and interleukin-10 (IL-10), or using contact-dependent suppression [48,49]. Numerous immune cells, including monocytes, macrophages, CD4^+^ T cells, and CD8^+^ T cells are inhibited by FOXP3^+^ Tregs. The large accumulation of these cells impairs the effector T cells’ ability to create an effective defense [48,49,50]. Also, they can use the metabolites produced by the tumor cells as an energy source, preferring the fatty acid energy supply pathway [51]. Consequently, the cancer cells multiply in an immune-suppressive environment where FOXP3^+^ Tregs are increased in number, while effector T cells and dendritic cells (DCs) activities become ineffective [52].

Patients with *Helicobacter (H.) pylori* infection express FOXP3^+^ Tregs at much higher levels than negative patients. Moreover, it has been discovered that *H. pylori* can modify the stomach microbiota, upregulating FOXP3^+^ Tregs, TGF-β, and IL-10 expression [53]. Additional findings showed that GC patients infected with *H. pylori* had higher levels of FOXP3^+^ Tregs cell infiltration in the local mucosa and peripheral blood mononuclear cells (PBMCs), the ratio of Tregs/Th17 and Th1/Th2 being disturbed. Furthermore, FOXP3^+^ Treg cells promote tumor progression by helping neoplastic cells escape from immunosurveillance by secreting TGF-β. At the same time, a high level of TGF-β and IL-6 in the TME stimulates the differentiation and expansion of FOXP3^+^ Treg cells [46,48,49]. As a result, FOXP3^+^ Tregs play a significant role in the genesis of precancerous lesions and GC [54,55].

Th17 cells are a subset of CD4^+^ T helper cells that secrete interleukin interleukin-17 (Il-17) with a pro-inflammatory effect. These cells in some malignancies promote tumor progression by amplifying inflammation or supporting tumor-promoting cytokines, while in others help to eliminate tumor cells by activating CTLs [56].

Th17 cell’s increased level was linked to advanced clinical stages in GC. Moreover, patients with GC with high IL-17 concentrations were found to have a bad prognosis [56,57,58,59]. Recent research has highlighted the importance of the disturbed balance between Th17 and FOXP3^+^ Tregs cells in GC. Patients with advanced GC have a greater Th17/FOXP3^+^ Tregs ratio than healthy controls [36,48]. Additionally, patients with lymph node metastases have shown a significantly elevated Th17/FOXP3^+^ Tregs cells ratio [46,60].

The apparent contradiction arises because GC progression is favored by the immunosuppressive TME created by FOXP3^+^ cells, while Th17 cells produce an inflammatory milieu. So, how can an immunosuppressive TME coexist with increased pro-inflammatory Th17 cells? Th17 cells produce IL-17, which causes stomach epithelial cells to produce interleukin-8 (IL-8), maintaining chronic inflammation. Prolonged inflammation may facilitate the gradual progression from chronic gastritis to premalignant gastric lesions [56,57]. Through interleukin-6 (IL-6), TGF-β, and interleukin-21 (IL-21), the TME stomach myofibroblasts stimulate Th17 differentiation, which by secretion of IL-17 will promote tumor progression [58,59]. On the other hand, we must consider the context-dependent functions of FOXP3^+^ Tregs, which not only support anti-tumor immunity but also contribute to maintaining the balance of immune responses in the TME. Tregs may help to limit excessive Th17 activity, which otherwise can lead to tissue damage, explaining how Tregs and Th17 coexist in the TME [60].

### 2.2. B Lymphocytes

B cells are becoming more widely recognized as essential parts of TILs. According to recent studies, B cells can constitute a substantial percentage of TILs. Through a variety of processes, such as the production of antibodies, the presentation of antigens, and the development of tertiary lymphoid structures that promote localized immune responses, B cells support the immune response to malignancies [61,62].

#### 2.2.1. Regulatory B Cells (Bregs)

Bregs with inhibitory phenotypes capable of anti-inflammatory cytokines production, such as IL-10, TGF-β, and interleukin-3 (IL-3) were identified. Nevertheless, these anti-inflammatory cytokines can accelerate the progression of malignancies [63,64]. Bregs express inhibitory molecules such as Fas Ligand (FasL) and PDL-1 and can support tumor progression by suppressing anti-tumor immune responses. In GC IL-10 and TGF-β produced by Bregs decreases CD4^+^ T cell functions and promotes FOXP3^+^ Tregs expansion. Inhibiting the differentiation into Th1 and stimulating differentiation into FOXP3^+^CD4^+^ Treg and Th2, IL-10 can additionally disrupt the delicate balance between Th1 and Th2 responses [65].

An analysis of patients with chronic gastritis revealed the number of Bregs in *H. pylori*-infected patients was significantly higher than in uninfected patients. This finding suggests that Bregs may be involved in modulating the inflammatory response to *H. pylori* infection. Still, when the patient enters the GC phase, these cells can favor the tumor progression [66].

#### 2.2.2. Tertiary Lymphoid Structures (TLSs)

TLSs are ectopic lymphoid structures that resemble secondary lymphoid organs which are formed in non-lymphoid tissues at the site of persistent inflammation. TLSs are composed of B cell aggregates within a follicular dendritic cell (FDC) meshwork. T cells and specialized blood vessels known as high endothelial venules surround the B cells. These structures are linked with increased tumor survival in GC [67]. TLSs are thought to be formed in the normal gastric mucosa because of chronic inflammation caused by a prolonged *H. pylori* infection [68].

The latest research revealed that the GC tissue contains aggregates of B cells, T cells, and FDC. Antigen-activated B cells often reach the GC tissue where they differentiate into GC B cells, which then differentiate into plasmablasts and remain active or become memory B cells within secondary lymphoid organs. TLSs are thought to function on the same basis. GC was found to contain nearly every stage of the B cells, including GC B cells, plasmablasts, and many memory B cells. Most B lymphocytes infiltrating GC are organized in TLSs, are sensitized to antigen, and have the capacity to differentiate into antigen-presenting cells (APCs) and multiply within TLSs, except the lymph nodes [69,70,71]. Furthermore, some studies suggest that tumor-infiltrating B cells can predominantly act as APCs. These APCs display antigens in a way that promotes T cell exhaustion, inhibiting CTLs and promoting FOXP3^+^ Tregs, enhancing cancer cells’ survival and tumor metastasis. Moreover, the tumor-infiltrating B cells can produce cytokines that can encourage a persistent inflammatory state in the TME, which promotes angiogenesis, metastasis, and tumor formation. At the same time, some tumor-infiltrating B cells can stimulate TAMs polarization into an M2 phenotype, while others produce antibodies to target tumor cells, which later form immune complexes that activate Fc receptors on immune cells, leading to the release of pro-tumorigenic factors. In conclusion, even if tumor-infiltrating B cells function as APCs and support anti-tumor immunity, they can cause immunosuppression, inflammation, and tissue remodeling, leading to tumor spread [72,73].

### 2.3. NK Cells

TILs also include NK cells, which are a crucial component of the immune system. These cells are necessary for identifying and destroying tumor cells, enabling a prompt immune response because their action is not condition by a previous contact with an antigen [74].

NK cells can identify and eliminate gastric tumor cells coated in antibodies by attaching to Fc region with their CD16 receptors. This triggers the release of cytotoxic granules containing perforin and granzymes, followed by apoptosis in the target cells [74,75]. Also, NK cells may induce apoptosis by releasing tumor necrosis factor-alpha (TNF-α) or by binding to their death receptors [74,76].

The Fas/FasL system plays an important role in the apoptosis of tumor-specific lymphocytes. The recent literature revealed that GC cells have a high proportion of NK cells expressing the Fas receptor, strongly correlated with an increased apoptosis rate of NK cells. As a result, patients with GC have a significantly higher rate of NK cell apoptosis compared to controls and a higher possibility of cancer progression. The apoptosis and Fas expression rate is even higher in NK cells from the gastric tumor tissue itself compared to circulating NK cells in the peripheral blood collected from the same patients [76,77,78].

Decreased NK cell activity was significantly associated with more advanced gastric tumor stages, such as larger size, vascular involvement, and lymph node metastases. Furthermore, the density of NK cells within the tumor was found to be an independent prognostic factor for overall and disease-free survival in GC. Similarly, a higher percentage of NK cells in peripheral blood correlates with longer survival and earlier cancer stages and can serve as an independent prognostic biomarker in GC [76,78].

NK Group 2 Member D (NKG2), an important receptor for the activation of NK cells, is significantly expressed in GC. When cancer cells express NKG2D ligands, they are more susceptible to NK cells. A study testing the susceptibility of GC cells to NK cells demonstrated that the samples with GC which expressed NKG2D ligands presented a better prognosis and decreased metastasis rate [79].

Prostaglandin E2 (PGE2), as a major enzymatic result of cyclooxygenase-2 (COX-2) overexpression, is involved in inflammation and tumor progression. Furthermore, according to the latest literature, the levels of NK cells within the tumor were negatively correlated with the expression of COX-2. On the other hand, PGE2 produced by GC cells suppressed the proliferation and increased the apoptosis of NK cells [80].

**Figure 1:**
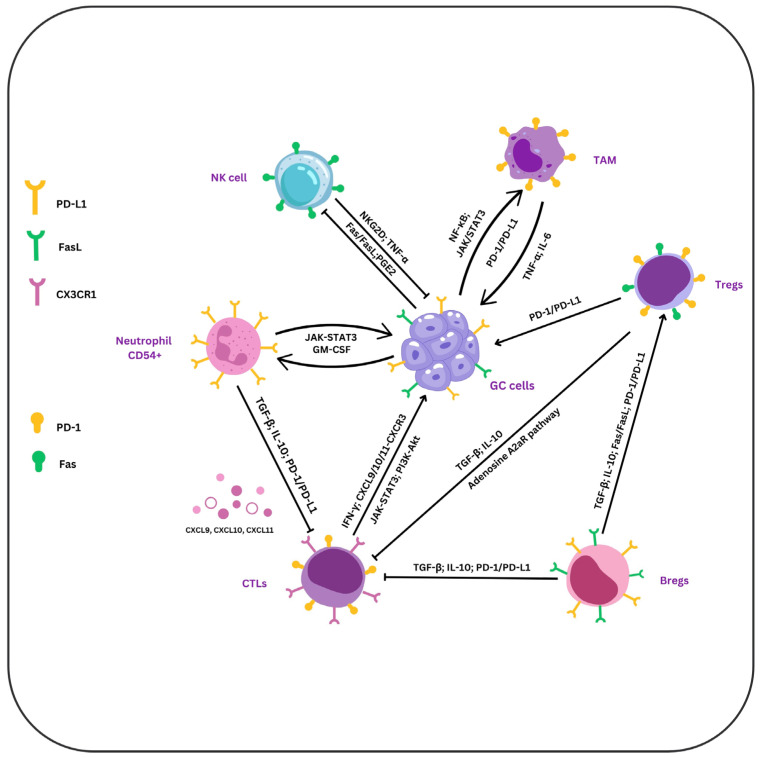
Immune cell dynamics in GC. TNF-α, IL-6, and signaling pathways (NF-κB and JAK/STAT3) drive the interaction between TAMs, GC, and NK cells, enhancing PD-L1 expression and promoting tumor growth. Through PD-L1 expression and release of IL-10 and TGF-β, Tregs inhibit Bregs and CTLs. They additionally help in immunological suppression, activating the A2AR and the Fas/FasL pathways, the last being involved in CTLs’ apoptosis. Using NKG2D and TNF-α release, NK cells induce GC cell apoptosis, whereas PGE2 secreted by the GC cells inhibits NK function. Also, NK cells use FasL to initiate GC cell destruction, whereas GC cells use Fas expression to stop their activity. In neutrophils, JAK/STAT3 signaling improves adhesion and activation, while through PD-1/PD-L1, TGF-β, and IL-10, they reduce CTLs’ function. Through their interaction with GC cells via IFN-γ, CTLs stimulate the synthesis of CXCL9, CXCL10, and CXCL11, attracting CTLs to the tumor site via CXCR3. Using PD-1/PD-L1 interactions and the release of TGF-β and IL-10, Bregs alter CTLs’ activity. By releasing immunosuppressive substances like TGF-β and VEGF and stimulating CTLs activation, PI3K-AKT signaling amplifies tumor growth and immune suppression. The JAK-STAT3 pathway in CTLs may reduce their cytotoxic effect and promote immunological tolerance.

Advanced combination therapy with adoptive NK cell therapy and immunoglobulin (Ig)G1 monoclonal antibodies was shown to induce a Th1-type immune response and decrease in peripheral Tregs, contributing to good tolerability and preliminary antitumor efficacy in GC. During the last few years, chimeric antigen receptor (CAR)-modified NK cells have been studied and demonstrated a promising immunotherapy approach for the cancer management. The ability of CAR-NK cells that are specific for CD19, CD20, epidermal growth factor receptor (EGFR), and human epidermal growth factor receptor-2 (HER-2) to kill target cells may be effective for HER-2^+^ GC [81,82]. Therefore, reversing NK cell dysfunction needs further investigation as a potential GC treatment Figure 1.

## 3. TAMs

In TME, monocytes in response to growth factors generated by tumors, stromal cells, and chemokines, differentiate into TAMs. In GC, these TAMs may promote genetic instability, support cancer stem cells, accelerate metastasis, and help create an immunosuppressive TME through the inhibition of T-cell activation [83,84].

Usually polarizing into either pro-inflammatory (M1) or anti-inflammatory (M2) phenotypes, TAMs are abundant in the TME. M2-polarized TAMs are predominant in GC and are linked to immunological suppression and tumor growth [83,84].

TAMs can contribute to an immunosuppressive milieu in GC that can reduce the CTLs’ function. This mechanism is mediated by PD-1/PD-L1 interaction on T cells, TAMs, and tumor cells, which decreases the cytotoxic activity of CTLs and promotes tumor growth. High PD-L1 expression in GC is associated with poor outcomes and with higher TAM levels. This suggests that TAMs might increase the immunosuppressive environment by stimulating tumor cells to produce PD-L1 [85].

**Figure 2:**
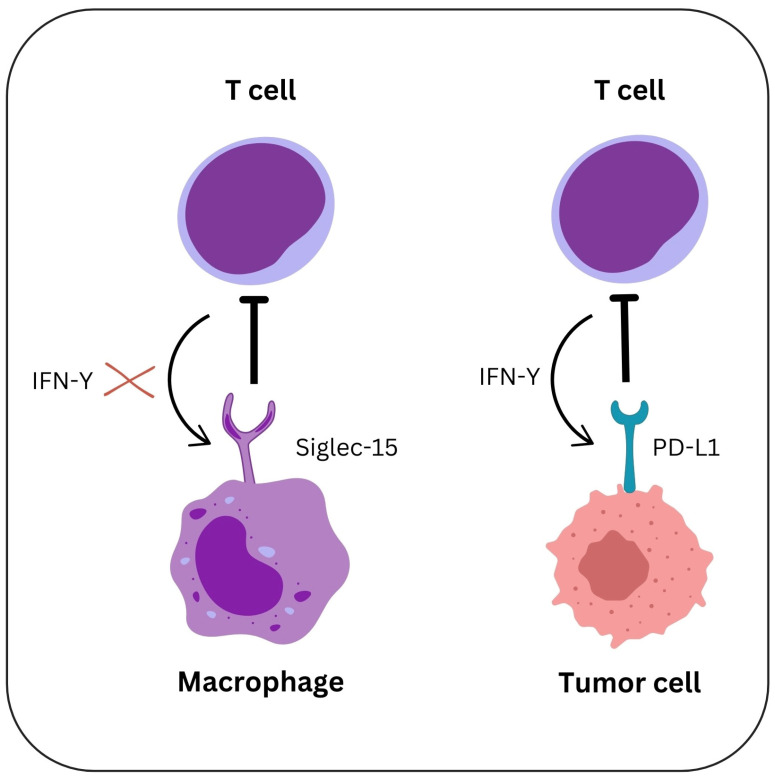
Mechanisms of PD-L1 and Siglec-15 in modulating T cell functions in the TME. The PD-L1 on GC cells and Siglec-15 on TAMs play complementary roles in inhibiting T-cell-mediated immunity. IFNγ from T cells induces upregulation of Siglec-15 expression on macrophages, thereby inhibiting their function. Similarly, tumor cells react to IFNγ by expressing PD-L1, which engages PD-1 on T cells, leading to T cell exhaustion.

Compared to M1 macrophages, M2-polarized TAMs, which are recognized for their pro-tumoral and immunosuppressive functions, express higher levels of Siglec-15. On TAMs, Siglec-15 expression can be increased by hypoxia, cytokines such as TGF-β, and signals from tumor cells. On TAMs, Siglec-15 inhibits the activity of CTLs. In contrast to PD-L1, which blocks T cells directly, Siglec-15 acts by modulating the immune suppression pathways mediated by TAMs. Siglec-15 increases the M2-like polarization of TAMs, helps in creating an immunosuppressive environment, and promotes tumor cell invasion, extracellular matrix remodeling, and angiogenesis. On the other hand, Siglec-15 can create a TME that inhibits the immune system by the synthesis of metabolites such as lactate and arginase [84,85,86] Figure 2.

**Figure 3:**
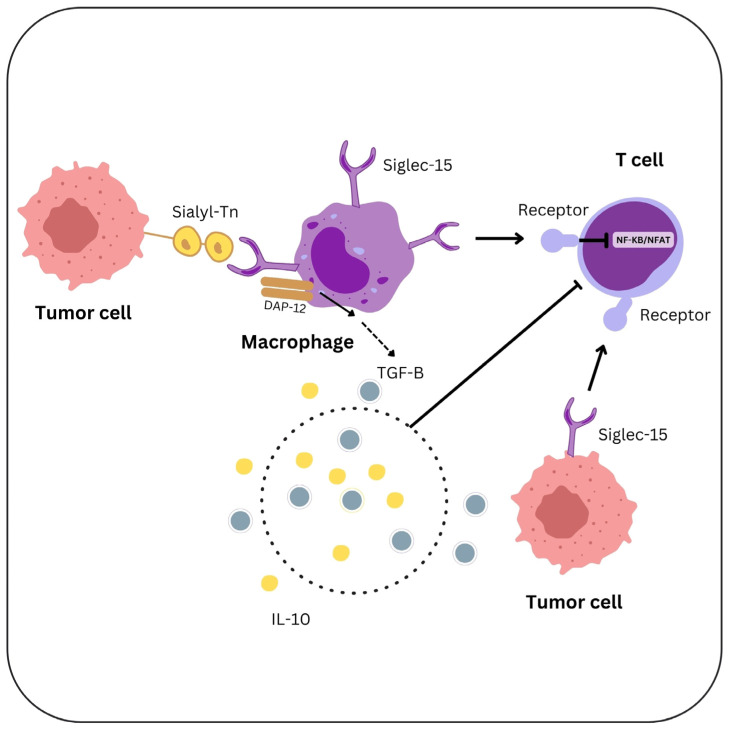
The influence of Siglec-15-expressing GC cells on TME Sialyl-Tn expressed by tumor cells bind to Siglec-15 on TAMs. Siglec-15 stimulates the release of TGF-β and IL-10 to decrease the T-cell responses and interacts with DAP12 to modify TAM activity. Siglec-15 also inhibits NF-κB/NFAT signaling, therefore reducing T-cell activation.

TAMs may be reprogrammed from an M2-like, immunosuppressive state to an M1-like, pro-inflammatory phenotype by Siglec-15 inhibitors or antibodies, as demonstrated in murine models. T-cell function may be restored, and anti-tumor immune responses may increase. Also, increased CTL infiltration and tumor growth inhibition were noted [87,88,89] Figure 3.

Additionally, a direct correlation between TAM levels in GC tissue and tumor vascularity, invasion capacity, nodal status, and clinical stage was found [90]. Therefore, GC may also benefit from a novel treatment approach that inhibits/TAM recruitment and survival in tumors.

Among the key players in the GC’s TME are MDSCs, a heterogeneous population of immature myeloid cells with important roles in immune regulation. In GC, these cells were correlated with cancer progression and metastasis. Regarding the pathophysiological mechanism, both TAMs and MDSCs are influenced by TGF-β and VEGF, which drive their recruitment and activation. TGF-β promotes the differentiation of monocytic MDSCs, increasing their suppressive activity, while VEGF facilitates their recruitment and activation. MDSCs exert an inhibitory effect on CTLs’ function while expanding the FOXP3^+^ Tregs population. Also, they release immunosuppressive factors (arginase-1, nitric oxide, reactive oxygen species) [91,92].

A better understanding of TAMs’ and MDSCs’ cooperative role in GC immunosuppression, both influenced by the same cytokine network, could contribute to identifying potential therapeutic agents targeting TGF-β and VEGF signaling pathways [93].

## 4. PD-1, PD-L1, and PD-L2

PD-1 (CD279), PD-L1, and Programmed Death-Ligand-2 (PD-L2) are members of the B7 family of cell-surface immune-regulatory proteins and crucial components of the immune checkpoint pathway, with an important role in anti-tumor immunity [94].

PD-1 is a T-cell immune checkpoint that suppresses autoimmunity and promotes immune tolerance in cells expressing PD-L1 (B7-H1; CD274) and PD-L2 (B7-DC). PD-1 is preferentially expressed on activated B cells, NK cells, and Tregs, all contributing to the immunosuppressive TME. PD-L1 is expressed on the surface of several different immune cell types and tumor cells. T cells that are activated on the PD-1/PD-L1 receptor develop peripheral immunologic tolerance [94,95]. Multiple solid tumors, including GC takeover this immune barrier by expressing PD-L1, which creates an immunosuppressive TME and prevents T-cell cytolysis [96]. PD-L2, a second ligand for PD-1, has a more restricted expression pattern, predominantly on dendritic cells, macrophages, and mast cells [94,95].

Oncogenic signaling can induce PD-L1 expression on solid tumor cells either using the phosphatidylinositol-3-kinase-protein kinase B (PI3KAKT) pathway or Janus kinase and signal transducer and activator of transcription (JAK-STAT) 3 signaling [97,98]. Interferon-gamma (IFN-γ), produced by TILs is one of the most potent PD-L inducers. It upregulates PD-L1 expression on tumor cells through the JAK-STAT3 pathway and impairs the cytotoxicity of CTLs against the cancer cells. Furthermore, in GC tissue samples, PD-L1 expression on tumor cells positively correlates with the presence of CTLs in the stroma and IFN-γ expression in the tumor, suggesting that PD-1/PD-L1 antagonists’ function better in GC patients whose TME contains a significant proportion of CTLs [99,100].

TAMs are another important TME component that can induce PD-L1 expression in GC cells by releasing pro-inflammatory cytokines (TNF-α, IL-6, IL-8) which activate the nuclear factor kappa-light-chain-enhancer of activated B cell (NF-κB) and STAT3 signaling pathways [101].

The CXCL9/10/11-CXCR3 signaling axis is induced by IFN-γ and is involved in attracting effector T cells (CTLs and Th1 cells), NK cells, and other immune cells to the TME. The excessive release of these chemokines can produce a pro-inflammatory environment, which can ironically help in tumor growth. When CXCR3 binds to its ligands (CXCL9/10/11), it initiates signaling pathways that promote immune cell migration and differentiation [102]. Chen-Lu Zhang et al. demonstrated that CXCL9/10/11-CXCR3 upregulates the expression of PD-L1 by activating the STAT3 and PI3K-Akt signaling pathways in GC cells [103]. Also, they found a significant positive correlation between the expression of PD-L1 and CXCR3 in GC patient tissues [103].

Neutrophils represent a large proportion of immune cells found in solid tumors, which exhibit different phenotypes according to the TME. Ting-ting Wang et al. showed that neutrophils with an activated and immunosuppressive phenotype (CD54^+^) enhance immunological suppression and the progression of GC through the GM-CSF-PD-L1 pathway [104]. The GC TME promotes the survival and activation of these neutrophils, with tumor-derived GM-CSF playing a key role in inducing PD-L1 expression on neutrophils through the JAK-STAT3 signaling pathway. These activated PD-L1-expressing neutrophils suppress T cell function in a PD-L1-dependent manner and are associated with disease progression and poor patient survival [104,105].

AT-rich Interactive Domain-containing protein 1A (ARID1A) acts as a tumor suppressor gene, its mutations being linked to PD-L1 upregulation [106]. A recent study examined the association between ARID1A loss and higher PD-L1 expression in a larger sample, showing a correlation with MSI-H and EBV status. In MSI-H GC, the degree of PD-L1 expression was even higher in tumors that had lost ARID1A [107].

PD-L1 expression in GC varies depending on the molecular subtype Table 1.

EBV-positive subtype has the highest rate of PD-L1 expression, both in tumor and immune cells in the TME [108]. Ruri Saito et al. found that PD-L1 was overexpressed in 34% of the cancer cells and 45% of the immune cells in EBV-associated GC. Also, PD-L1 expression in this subtype was associated with diffuse histology and deeper tumor invasion [109]. A meta-analysis studying the prognostic significance of PD-L1 in GC found that PD-L1 expression is a valuable predictor of prognosis as it is associated with shorter overall survival, higher T stage, and lymph node metastasis [110]. MSI GC also demonstrates a relatively high PD-L1 expression, this subtype being associated with a higher mutational burden activation, with PD-L1 expression particularly in immune cells [111,112]. In the GS GC, the PD-L1 expression is lower; some positivity may be seen in immune cells. In the CIN subtype, the expression is variable, usually in immune cells. As a result, EBV-positive and MSI GC patients are prime candidates for PD-1-directed therapy [112].

## 5. Siglec-15

**Figure 4:**
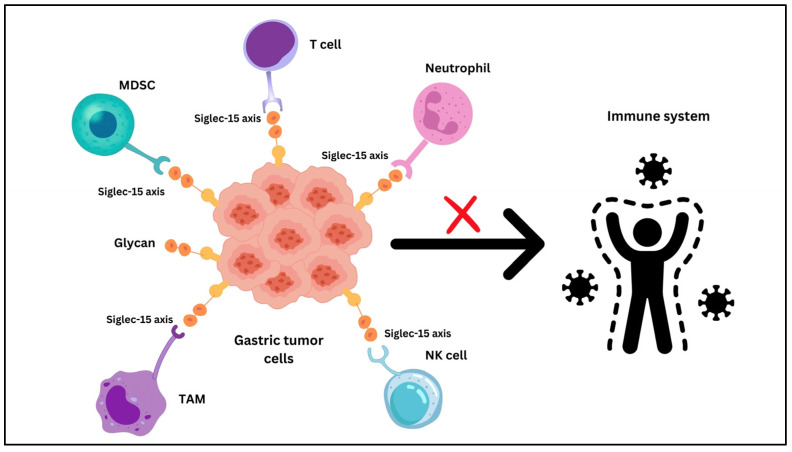
The role of Siglec-15 in shaping immune responses in GC MDSCs, neutrophils, NK cells, TAMs, and other immune cells have Siglec-15 receptors that are bound by sialylated glycans expressed by gastric tumor cells. By inhibiting T-cell function and promoting tumor growth, these interactions result in immune evasion.

Siglec-15 is an immunomodulatory protein that gained interest due to its function in the immune system, especially in cancer therapy [113]. Like PD-L1, Siglec-15 is a part of immune evasion strategies malignancies employ to decrease the immune response [114] Figure 4.

Even though anti-PD-1/PD-L1 is now the most effective immunotherapy for cancer treatment, most patients develop natural or acquired resistance. Other immune inhibitory mechanisms may be operating in conjunction with or alongside PD-1/PD-L1 inhibition in these resistant patients, offering novel immunological targets that could potentially increase the efficacy of cancer immunotherapy [94].

Siglec-15 is one of the Siglec gene family members with a sialic acid-binding immunoglobulin-type lectin structure. It consists of two Ig-like domains, a transmembrane domain with a lysine residue, and a short cytoplasmic tail, binding preferentially sialyl-Tn (sTn) [87]. Normally, Siglec-15 mRNA is very low in most immune cell types and steady-state normal human tissues, being detected on macrophages and/or dendritic cells of the human spleen and lymph node [87,115]. Still, it is widely increased in human cancer cells and/or TAMs/myeloid cells, in contrast to its minimal expression level on macrophages in normal tissues. When compared to the corresponding normal tissues, it is predominantly upregulated in colon, endometrioid, and thyroid tumors and highly expressed in bladder, kidney, lung, and liver malignancies [116,117].

Siglec-15 also shows high homology with B7 family members, and it is considered a macrophage-associated T-cell immunosuppressive molecule [85]. In a recent paper analyzing the role of Siglec-15 in the suppression of T cell activity using various assays in both humans and mice, authors showed the suppressive role of Siglec-15 on antigen-specific T cell response in vivo, which is dependent on IL-10. However, in contrast to PD-L1 expression, Siglec-15 expression was inhibited by IFN-γ in vitro [87].

Also, it was demonstrated that in human non-small cell lung cancer (NSCLC), Siglec-15 expression was mutually exclusive with PD-L (B7-H1), partially due to its induction by M-CSF and downregulation by IFN-γ [11,87,118]. Moreover, macrophage-specific knockout of Siglec-15 in mice enhanced T cell-mediated anti-tumor immunity and slowed the tumor’s growth. In the same study, anti-Siglec-15 monoclonal antibodies (mAbs) inhibited the growth of tumors and reduced the inhibitory effects of Siglec-15 on T cells [87].

Immunosuppressive or “cold” tumors, which often show low levels of immune cell infiltration, express high Siglec-15 levels in the TME. This implies that Siglec-15 contributes to immunological tolerance maintenance and immune response suppression by encouraging immunological evasion and preventing immune cell activation, like CTLs [87,113].

Siglec-15 can still be expressed on immune cells in the TME, especially on TAM and MDSCs, even if it is linked to a non-inflamed TME. Depending on the tumor, these immune cells may be “educated” to promote immunosuppression, and Siglec-15 might contribute to immunological escape by reducing the activity of CTLs, as discussed previously [113,114,115,116].

Given their different ways of action and complementary functions in immune system modulation, anti-Siglec 15 antibodies show promise as an adjuvant treatment to anti-PD-L1, especially in non-inflammatory (“cold”) tumors or later in the tumor’s development, when the PD-L1 pathway may not be as prominent. Therefore, anti-PD-L1 and anti-Siglec 15 therapies together may target immune evasion mechanisms at various tumor growth phases [12,116] Table 2.

The development of small-molecule inhibitors that target Siglec-15 is supported by the work of Zhang et al., which discovered SHG-8 as a novel small-molecule inhibitor of the Siglec-15-sialic acid axis in colorectal cancer [120]. The potential for developing small-molecule inhibitors has also been demonstrated by studies on other Siglec family members. For example, research on Siglec-8 resulted in the production of inhibitors that modify the activity of immune cells [118]. According to these findings, small-molecule inhibitors show promise as cancer immunotherapy agents, especially in tumors where conventional immune checkpoint inhibitors may not be effective [119,120].

Several trials targeting Siglec-15 are ongoing. In one of them, the mAbs NC318 is assessed alone or in combination with pembrolizumab in patients with advanced or metastatic NSCLC [121,122]. PYX-106, another novel blocking mAbs that targets Siglec-15, is being studied in a phase I clinical trial that includes patients with advanced solid tumors [123].

In GC, Siglec-15 expression was associated with histological classification, angiolymphatic invasion, and surgical staging [124,125]. However, the current literature on Siglec-15 expression in GC remains limited, and it is unknown if the mutual exclusivity between Siglec-15 and PD-L1 expression observed in NSCLC or other tumors can be extended to GC. Moreover, there is a lack of comprehensive information regarding the expression of Siglec-15 on different molecular subtypes of GC [122,123,124,125]. Further research is necessary to determine if similar regulatory mechanisms, immune evasion strategies, and checkpoint inhibition are applied to GC TME.

## 6. Conclusions

In summary, the complex interactions between Siglec-15, PD-L1, and TILs in the TME of GC reveal a dynamic and complex tumor immune milieu. Anti-tumor immunity and patient outcomes depend significantly on TILs density. On the other hand, the overexpression of immune checkpoints such as PD-L1 and Siglec-15 may compromise immune defense mechanisms resulting in treatment resistance.

The diversity of expression patterns and functions of PD-L1 and Siglec-15, along with the density and activity of TILs indicate that their global assessment may allow a better understanding of the immunological landscape diversity in GC. This may open the door to more individualized and successful immunotherapy approaches.

Future investigations should focus on the underlying processes of co-expression and interaction of these markers and their influence on clinical outcomes. Applying advanced immunohistochemistry with molecular pathology tools like next-generation sequencing and spatial transcriptomics would allow a better understanding of these relationships and generate new immunotherapies combinations that favorably impact GC’s response and prognosis.

## Figures and Tables

**Table 1:** PD-L1 expression in GC according to molecular subtypes.

Characteristics	EBV-Positive GC	MSI GC	GS GC	CIN GC
Molecular Features	✓ presence of EBV within the tumor cells ✓ high levels of DNA hypermethylation ✓ frequent PD-L1 and PD-L2 overexpression [3,4,5,108,109]	✓ ↑ frequency of mutations, particularly in repetitive DNA microsatellites, due to defects in the DNA MMR system [3,4,5,110,111,112]	✓ absence of significant chromosomal alterations ✓ often associated with diffuse-type histology, including the presence of signet ring cells [3,4,5,111,112].	✓ extensive chromosomal alterations, including amplifications of oncogenes like HER-2 [3,4,5,111,112].
Immune Cell Infiltration	✓ ↑ level of immune cell infiltration, particularly with T cells (CTLs and NK cells) [3,4,5,108,109]	✓ ↑ mutation burden ⇒ production of numerous neoantigens, recognized as foreign by the immune system ⇒ ↑ TILs, particularly CTLs cells [3,4,5,110,111,112]	✓ ↓ level of immune cell infiltration ✓ ↑ immunosuppressive TME, with ↑ Tregs and MDSCs ⇒ inhibits effective immune responses [3,4,5,111,112].	✓ variable immune cell infiltration, often lower than in MSI and EBV-positive subtypes [3,4,5,111,112].
Immunotherapy Response	✓ good candidates for PD-1/PD-L1 inhibitors [3,4,5,108,109]	✓ good candidates for PD-1/PD-L1 inhibitors [3,4,5,110,111,112]	✓ ↓ response to immunotherapy [3,4,5,111,112].	✓ if HER-2 overexpression ⇒ HER2-targeted therapies ✓ ↓ response to immunotherapy unless they also exhibit ↑ levels of immune checkpoint markers [3,4,5,111,112].

**Table 2:** Siglec-15 expression in GC.

Characteristic	Observation
Expression Levels	✓ SIGLEC-15 expressed in various human cancers, including GC ✓ Expression has been associated with poor outcomes in solid tumors [113]
TME	✓ High SIGLEC-15 expression—linked to a non-inflamed TME (“cold tumors”) and contributes to tumor immune evasion [113,114,115,116]
Prognostic Significance	✓ ↑ SIGLEC-15 expression correlates with advanced tumor stages => possible prognostic marker in GC [87,113]
Immune Cell Infiltration	✓ ↑ SIGLEC-15 expression is associated with TAMs and MDSCs => the immune response modulation within the TME, inhibiting the activity of CTLs [13,87,89]
Immunotherapy Response	✓ Targeting SIGLEC-15 => novel immunotherapeutic approach for patients who do not respond to PD-1/PD-L1 inhibitors [119,120,121,122,123,124,125]

## Abbreviations

**A2AR**—Adenosine A2A Receptor
**APC**—Antigen Presenting Cell
**ARID1A**—AT-rich Interactive Domain-containing protein 1A
**Bregs**—B Regulatory Cells
**CAR NK**—Chimeric Antigen Receptor Natural killer cells
**CIN**—Chromosomal Instability
**CTLs**—Cytotoxic T Lymphocytes
**CXCL9, CXCL10, and CXCL11**—C-X-C Motif Chemokine Ligand 9, 10, and 11
**CXCR3**—C-X-C Motif Chemokine Receptor 3
**DAP12**—DNAX-Activation Protein of 12 kDa
**DCs**—Dendritic Cells
**DNA**—Deoxyribonucleic Acid
**EBV**—Epstein–Barr Virus
**EGFR**—epidermal growth factor receptor
**Fas/FasL**—Fas/Fas Ligand
**FDC**—follicular dendritic cell
**FOXP3**—Forkhead Box Protein P3
**GC**—Gastric Cancer
**GS**—Genomic Stable
***H. pylori***—*Helicobacter pylori*
**HER-2**—Human Epidermal Growth Factor Receptor 2
**HLA**—Human Leucocyte Antigen
**IFN-γ**—Interferon-gamma
**Ig**—Immunoglobulin
**IL-10**—Interleukin-10
**IL-17**—interleukin-17
**IL-21**—interleukin-21
**IL-6**—Interleukin-6
**IL-8**—Interleukin-8
**JAK/STAT3**—Janus Kinase/Signal Transducer and Activator of Transcription 3
**mAbs**—monoclonal antibodies
**MDSCs**—Myeloid-Derived Suppressor Cells
**MHC**—major histocompatibility complex
**MMR**—Mismatch Repair
**MSI**—Microsatellite Instability
**NF-κB/NFAT**—Nuclear Factor kappa-light-chain-enhancer of activated B cells/Nuclear Factor of Activated T-cells
**NK**—Natural Killer
**NKG2D**—Natural Killer Group 2 Member D
**NSCLC**—non-small cell lung cancer
**PD-1**—Programmed Cell Death-Protein-1 receptor
**PD-L1**—Programmed Cell Death Ligand-1
**PD-L2**—Programmed Cell Death Ligand-2
**PGE2**—Prostaglandin E2
**PI3K-AKT**—Phosphoinositide 3-Kinase-Protein Kinase B
**RA**—Rheumatoid Arthritis
**Sialyl-Tn**—Sialylated Tn Antigen
**Siglec-15**—Sialic Acid-Binding Immunoglobulin-Like Lectin 15
**TAMs**—Tumor-Associated Macrophages
**TCR**—T Cell Receptor
**TGF-β**—Transforming Growth Factor-beta
**Th**—T helper
**TILs**—Tumor-Infiltrating Lymphocytes
**TLSs**—Tertiary lymphoid structures
**TME**—Tumor Microenvironment
**TNF-α**—Tumor Necrosis Factor-alpha
**Tregs**—Regulatory T Cells
**TRM**—Tissue-Resident Memory
**VEGF**—Vascular Endothelial Growth Factor
